# A Novel Peptide Derived from *Arca inflata* Induces Apoptosis in Colorectal Cancer Cells through Mitochondria and the p38 MAPK Pathway

**DOI:** 10.3390/md20020110

**Published:** 2022-01-29

**Authors:** Chunlei Li, Sirui Zhang, Jianhua Zhu, Weijuan Huang, Yuanyuan Luo, Hui Shi, Dongbo Yu, Liguo Chen, Liyan Song, Rongmin Yu

**Affiliations:** 1 Biotechnological Institute of Chinese Materia Medica, Jinan University, 601 Huangpu Avenue West, Guangzhou 510632, China; lichunlei@jnu.edu.cn (C.L.); tzjh@jnu.edu.cn (J.Z.); yyluooo@163.com (Y.L.); sh613050@163.com (H.S.); 2 Integrated Chinese and Western Medicine Postdoctoral Research Station, Jinan University, Guangzhou 510632, China; 3 Department of Pharmacology, Jinan University, 601 Huangpu Avenue West, Guangzhou 510632, China; zhangsiruijnu@126.com (S.Z.); wjhuang@jnu.edu.cn (W.H.); 4 Department of Natural Product Chemistry, Jinan University, 601 Huangpu Avenue West, Guangzhou 510632, China; 5 Department of Cardiovascular Care, ThedaCare Regional Medical Center, Appleton, WI 54911, USA; dongbo.yu@thedacare.org; 6 Institute of Integrated Chinese & Western Medicine, Jinan University, 601 Huangpu Avenue West, Guangzhou 510632, China

**Keywords:** *Arca inflata*, antitumor peptide, apoptosis, MAPK signaling, calmodulin, colorectal cancer

## Abstract

Colorectal carcinoma (CRC) is one of the major causes of cancer-related incidence and deaths. Here, we identified a novel antitumor peptide, P6, with a molecular weight of 2794.8 Da from a marine Chinese medicine, *Arca inflata* Reeve. The full amino acid sequence and secondary structure of P6 were determined by tandem mass de novo sequencing and circular dichroism spectroscopy, respectively. P6 markedly inhibited cell proliferation and colony formation, and induced apoptosis in CRC cells. Mechanistically, transcriptomics analysis and a serial functional evaluation showed that P6 induced colon cancer cell apoptosis through the activation of the p38-MAPK signaling pathway. Moreover, it was demonstrated that P6 exhibited antitumor effects in a tumor xenograft model, and induced cell cycle arrest in CRC cells in a concentration-dependent mode. These findings provide the first line of indication that P6 could be a potential therapeutic agent for CRC treatment.

## 1. Introduction

Colorectal cancer (CRC) is by far the third most common malignant tumor, and it ranks as the second leading cause of death from cancer in the world [1]. CRC deaths have ameliorated over the past few decades on account of headways in both early diagnosis and intervention; however, long-term declines in mortality have slowed during last five years [2,3]. At an early stage, the five-year survival rate of CRC is higher than 80%; nevertheless, it decreases to approximately 10% at the advanced stages [1]. Therefore, it is urgent to develop effective anti-CRC agents with low toxicity to effectively treat and prevent the recurrence and metastasis of CRC.

The ocean is home to most of the world’s life. Diverse marine organisms live and thrive in an aquatic buffer system despite the many extreme physiologic stresses, such as high salinity, high pressure, and extreme hypoxia. Living in such a special environment, many marine organisms have evolved with special adaptions and have become an affluent source of bioactive substances with broad health benefits [4]. Due to the specific environment in which marine organisms live, the structure of marine antitumor peptides is very different from that of terrestrial animal and plant peptides. Most of them are small molecular cyclic or linear peptides that are rich in polar amino acids, D-type amino acids, hydroxyl acids, thiophenol, and oxazole rings. Some also contain alkene and alkyne bonds, which greatly improve the biological stability and bioavailability of the peptides. Didemnin B is a phenolic peptide compound isolated from the tunicate *Trididem numsolidum* from the Caribbean [5]. It has been reported that the real source of didemnin B may be cyanobacteria symbiotic with the tunicates. Didemnin B can directly bind to palmitoyl protein thioesterase, and exhibits strong activity in multiple tumor models [6]. Pettit et al. isolated a pentapeptide, dolastatin 10, from *Dolabella auricularia*. Dolastatin 10 is a secondary metabolite of the cyanobacteria *Sym ploca*, symbiotic with the sea hare [7]. Dolastatin 10 has excellent antitumor activity and can strongly inhibit the binding of vinblastine to tubulin noncompetitively (Kd = 1.41 μM) and can greatly affect microtubule assembly and microtubule-dependent guanosine triphosphate hydrolysis [8]. In addition, Dolastatin 15 is a cyanobacterial polypeptide isolated from the sea hare *D. Auricularia* from the Indian Ocean. It is a linear peptide containing seven amino acids or hydroxyl acid residues. Unlike dolastatin 10, dolastatin 15 binds directly to the vinblastine binding site of tubulin to exhibit prominent antitumor efficacy [9]. Moreover, natural peptides derived from marine organisms, some of which have been shown to act as signaling/regulatory molecules in various physiological processes, also exhibit potential antitumor effects [10]. Among them, lysine-rich peptides have shown selective cytotoxicity against both microbes and cancer cells [11]. Several studies have demonstrated that lysine-rich peptides have modes of action involving cellular uptake, mitochondrial apoptosis, and other intracellular events [12,13,14]. However, the mechanisms behind the cytotoxic and antitumor effects of lysine-rich peptides remain unclear.

*Arca inflata* Reeve is a bivalve mollusk of the Arcidae family that prevails along the coastlines of Asia. The farming production of *A. inflata* reaches up to 350,000 tons per year in China. Furthermore, *A. inflata* is a traditional marine Chinese medicine that has several therapeutic functions, including in cancer treatment. Previously, we identified two antitumor polypeptides and a glucanase from *A. inflata* [15,16,17]. However, the antitumor mechanism in *A. inflata*-derived peptides remains insufficiently understood. In this study, we found that a new lysine-rich peptide, P6, displayed potent anticancer activity towards HT-29 and DLD-1 colorectal cancer cells. P6 induced Ca^2+^ overload, ROS production, and mitochondrial dysfunction, thereby triggering apoptosis through the activation of the p38-MAPK signaling pathway. Moreover, P6 exerted antitumor effects in a tumor xenograft model.

## 2. Results

### 2.1. Identification and Physicochemical Properties of P6

**Figure 1:**
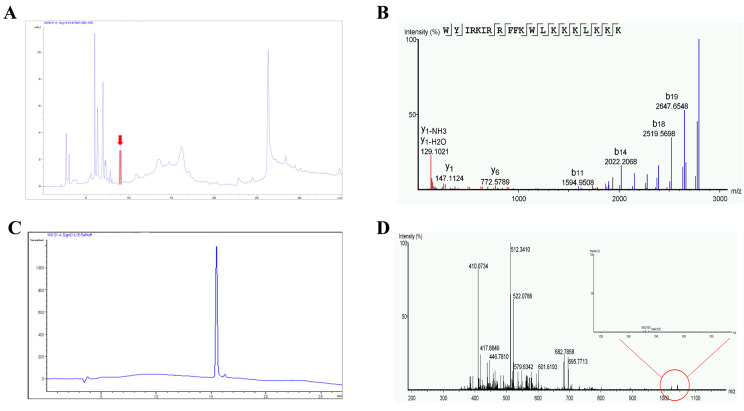
Purification and identification of peptide P6 from the hemolymph of *A. inflata*. (**A**) Isolation of native P6 (red arrow) from the pooled protein fraction by a C18 RP-HPLC column. (**B**) nanoESI-MS/MS analysis and sequencing of P6. (**C**) The purity of P6. (**D**) The precise molecular weight of P6 determined by ESI-MS.

**Figure 2:**
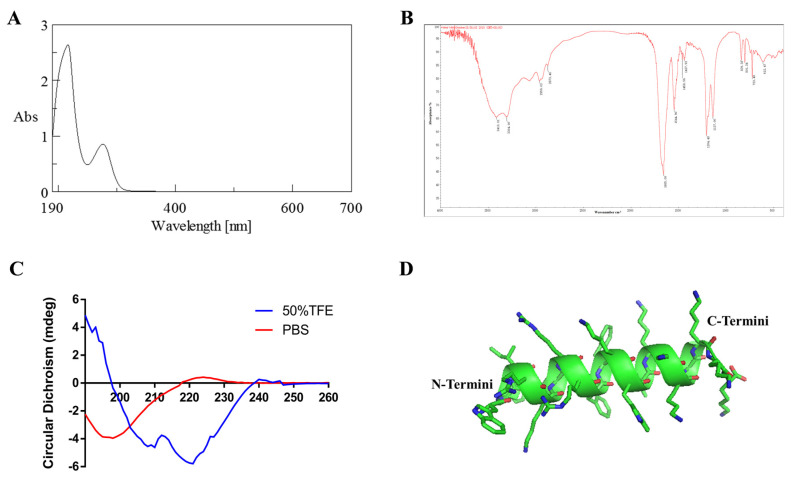
Physicochemical properties of P6. (**A**) The ultraviolet spectrum of P6. (**B**) The infrared spectrum of P6. (**C**) The circular dichroism spectrum of P6. (**D**) The three-dimensional structure of P6. The structure of P6 is shown as a merged cartoon and stick pattern. Green represents carbon atoms, blue represents nitrogen atoms, and red represents oxygen atoms.

*A*. *inflata* is a traditional marine Chinese medicine that has shown good efficacy in CRC treatment according to historical records in China. In our previous research, we were able to purify two polypeptides with moderate anti-neoplastic activity [15,16]. To further understand the antitumor constituents in *A. inflata* and their underlying anti-CRC mechanisms, we tracked the molecules that cause antitumor activity using peptide purification coupled with cytotoxicity experiments (Figure 1A). A peptide named P6 with a molecular weight of 2794.8 Da (Figure 1D) was subsequently identified from the hemolymph of *Arca inflata* (Figure 1C). The primary sequence of P6 was obtained as WYIRKIRRFFKWLKKKLKKK by de novo sequencing (Figure 1B). The sequence of P6 is rich in lysine residues and shows no resemblance to any known animal-derived anticancer peptides when searched in non-redundant protein databases via the NCBI BLASTP server. The physicochemical properties of P6 were also tested (Figure 2). The UV-vis absorption spectrum of P6 clearly showed a powerful absorption peak at approximately 210 nm and a shoulder peak at 280 nm, which are characteristic absorption peaks of the amido linkage of in the peptide primary structure and the aromatic amino acid residues of P6, respectively (Figure 2A). FT-IR spectroscopy was further applied to provide information about the conformational characterization of the peptides. As depicted in Figure 2B, two relatively strong bands that symbolizing amide I and amide II were observed at absorption frequencies of 1655.00 and 1544.90 cm^−1^, respectively, in the FT-IR spectrum. The amide I and amide II absorption distribution in P6 fits the standard bands for peptides of the amide I band, found between 1600 to 1700 cm^−1^ (C=O stretch), and the amide II band, found at approximately 1550 cm^−1^ (C=N stretch coupled with the N–H bending form) [18]. Additionally, absorption band frequencies at about 1650 and 1540 cm^−1^ are regularly recognized as the helical structure of peptides [15,19]. The characteristic absorption at 1655.00 and 1544.90 cm^−1^ implies that P6 consists of a high ratio of helical secondary conformation [15,20]. The CD spectrum of P6 was also detected. As shown in Figure 2C, P6 exhibited two positive peaks at 193 and 196 nm, as well as two negative ellipticity signals at 210 and 220 nm, which are a canonical peculiarity of peptide helical conformation. The three-dimensional structure of P6 was predicted as well (Figure 2D). The structure was compact with good amphipathicity, which results in a positively-charged hydrophilic surface and a hydrophobic surface (Figure 2D). 

### 2.2. P6 Inhibits Human CRC Cell Growth

**Figure 3:**
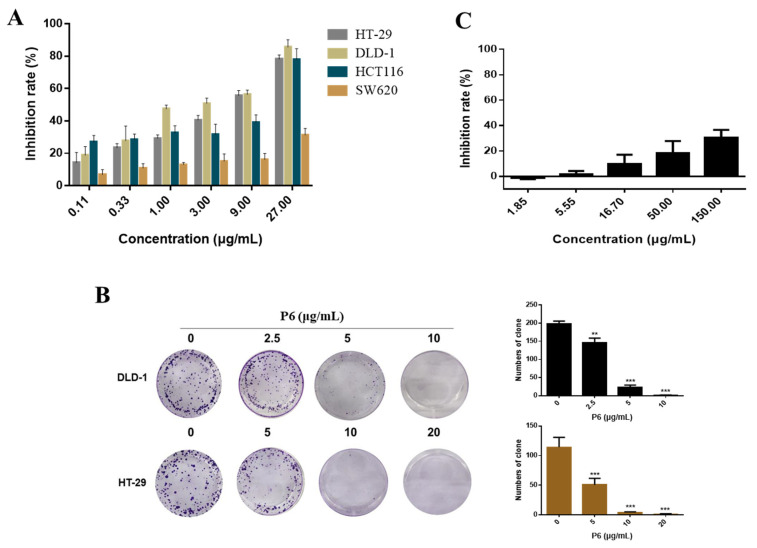
In vitro cytotoxicity of P6. (**A**) The inhibition rates of HT-29, DLD-1, SW620, and HCT116 cells treated with P6 for 48 h were determined by MTT assay. (**B**) Statistical histogram of the results from the colony formation assay used to investigate the inhibitory effect of P6 on the colony formation of HT-29 and DLD-1 cells. Each value is the average (±SEM) of triplicate samples. ** *p* < 0.01 and *** *p* < 0.001, compared with control. (**C**) The effect of P6 on the viability of human normal liver cells (L02).

To assess the inhibitory effect of P6 on the proliferation of human CRC cells, an MTT assay was performed. Several CRC cell lines—HT-29, HCT116, SW620, and DLD-1—were treated with serial concentrations of P6 for 48 h. P6 markedly decreased cell viability in a concentration-dependent manner in the tested CRC cells (Figure 3A). Among these cell lines, we chose the most sensitive cell lines, DLD-1 and HT-29, with IC_50_ values of 2.14 ± 0.28 μg/mL and 4.43 ± 0.15 μg/mL, respectively, for further analysis (Table 1). For better comparison and investigation of the underlying anti-CRC mechanism of action of P6, we used 0, 5, 10, and 20 μg/mL of P6 for HT-29 cells and 0, 2.5, 5, and 10 μg/mL of P6 for DLD-1 cells in further studies, according to the IC_50_ values. Both the serial concentrations used in HT-29 and DLD-1 cell lines included two of the same concentrations (5 and 10 μg/mL). Hence, it is still feasible to make a comparison between these two tested cell lines. Meanwhile, P6 exhibited a weak effect on human normal liver cells (L02; Figure 3C), suggesting that P6 is selective towards cancer cells. Moreover, the long-term inhibitory effects of P6 on HT-29 and DLD-1 cell proliferation were investigated through a colony formation assay. As shown in Figure 3B, P6 inhibited the proliferation of HT-29 and DLD-1 cells in a concentration-dependent manner, which confirmed the results of the MTT assay.

### 2.3. P6 Induces Apoptosis and Cell Cycle Arrest in HT-29 and DLD-1 Cells

**Figure 4:**
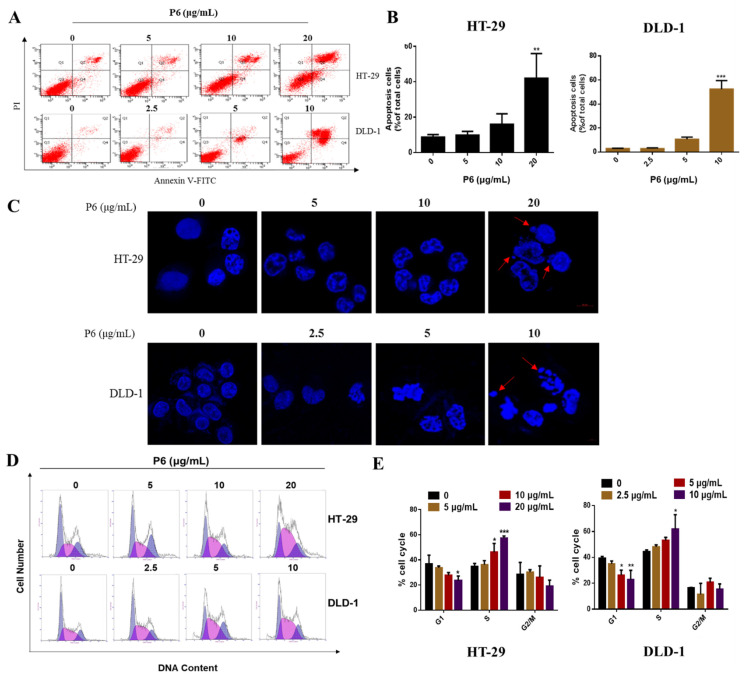
P6 induced cell apoptosis and cell cycle arrest in HT-29 and DLD-1 human CRC cells. (**A**) The pro-apoptotic effect of P6 on HT-29 and DLD-1 cells was analyzed by Annexin V-FITC/PI staining. (**B**) The Annexin V/PI staining of HT-29 and DLD-1 cells was quantified and displayed as a histogram. (**C**) Changes in HT-29 and DLD-1 cells treated with P6 were detected by Hoechst 33342 staining (×40 magnification). The red arrows indicate apoptotic cells. (**D**) P6 altered the cell cycle distribution in the CRC cells HT-29 and DLD-1. The cell cycle distribution after treatment with serial concentrations of P6 for 48 h in the HT-29 and DLD-1 cells was analyzed by FACS. (**E**) Quantitative histogram of HT-29 and DLD-1 cell cycle phases. Each value is the average (± SD) of triplicate samples. * *p* < 0.05, ** *p* < 0.01, and *** *p* < 0.001, compared with the control.

In order to detect whether P6 induced apoptosis, an important type of programmed cell death, CRC cells were treated with P6 for 48 h and then stained with Annexin V/PI and measured by flow cytometry. P6 produced a concentration-dependent induction of apoptosis in HT-29 and DLD-1 cells (Figure 4A,B). Subsequently, Hoechst 33342 staining was performed to detect nuclear fragmentation and apoptotic body production in the HT-29 and DLD-1 cells. As shown in Figure 4C, the apoptotic HT-29 and DLD-1 cells increased with P6 treatment at serial concentrations and were accompanied by the production of apoptotic bodies. 

Cell cycle progression is closely related to cancer cell proliferation. To investigate the mechanism by which P6 was prompting CRC cell apoptosis, we used flow cytometry to determine the effect of P6 on the CRC cell cycle distribution. As shown in Figure 4D, P6 treatment induced a marked cell cycle arrest at the S/G2 transition in a concentration-dependent manner in both HT-29 and DLD-1 CRC cells. Subsequently, 10 μg/mL of P6 caused nearly 50% of the CRC cells to arrest in S phase (Figure 4E). These results indicated that P6 could inhibit the proliferation of CRC cells through inducing cell cycle arrest at the S phase.

### 2.4. P6 Induces Mitochondrial Apoptosis and Boosts Ca^2+^ Influx in HT-29 and DLD-1 Cells 

**Figure 5:**
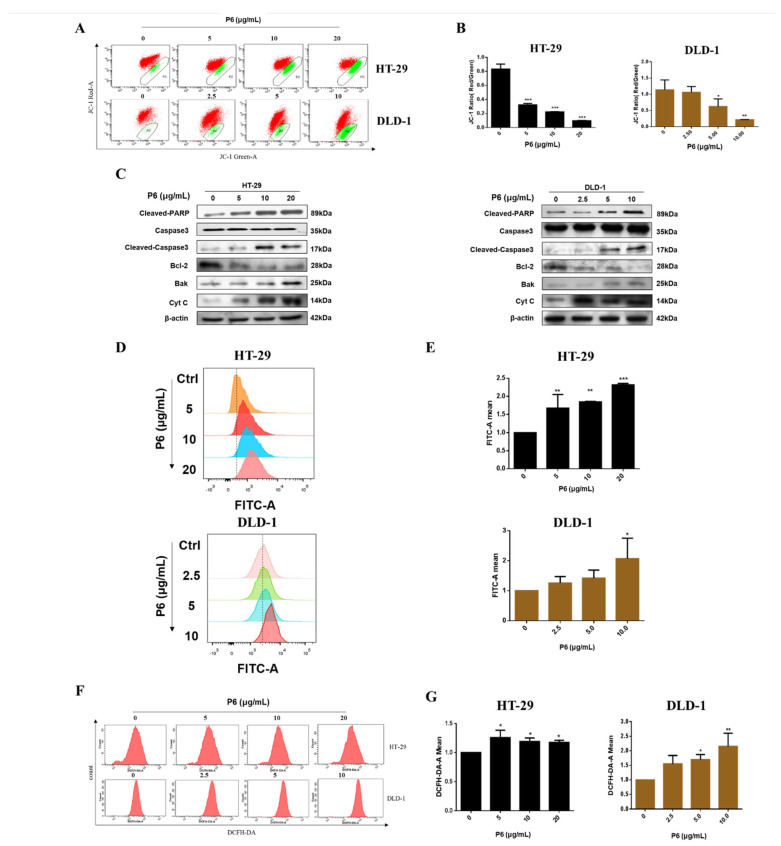
P6 induced cell apoptosis by the mitochondrial pathway and boosted Ca^2+^ influx in CRC cells. (**A**) Mitochondrial membrane potential of HT-29 and DLD-1 cells induced by different concentrations of P6. Red fluorescence and green fluorescence imply that the mitochondrial membrane potential is normal and decreased, respectively. (**B**) The mitochondrial membrane potential of HT-29 and DLD-1 cells was quantified and displayed as a histogram. (**C**) Changes in the expression levels of apoptosis-related proteins in HT-29 and DLD-1 cells after P6 treatment for 24 h were determined by Western blotting. (**D**) Effects of P6 on Ca^2+^ influx in HT-29 and DLD-1 cells. (**E**) The Ca^2+^ influx in HT-29 and DLD-1 cells was quantified and displayed as a histogram. (**F**) Effects of P6 on ROS of HT-29 and DLD-1 cells. (**G**) ROS of HT-29 and DLD-1 cells were quantified and displayed as a histogram. Each value is the average (±SD) of triplicate samples. * *p* < 0.05, ** *p*< 0.01 and *** *p* < 0.001, compared to the control group.

In order to further investigate the underlying mechanism of P6-induced apoptosis in HT-29 and DLD-1 cells, we further examined whether P6 induced apoptosis through arousing the mitochondrial pathway. JC-1 staining was applied to determine the mitochondrial membrane potential (MMP) changes in CRC cells. As demonstrated in Figure 5A,B, with the increase in apoptotic cells induced by P6, the green fluorescence of FITC-A also gradually increased. P6 induced marked MMP changes in a concentration-dependent manner in both CRC cell lines. By contrast, P6 showed an improved effect on HT-29 cells compared to DLD-1 cells. Western blotting detection verified the results of the mitochondrial membrane potential measurements. As depicted in Figure 5C, we found that P6 increased the expression levels of apoptosis-related proteins, including cleaved PARP and cleaved caspase-3, as well as the pro-apoptotic proteins Bak and Cyt C in a concentration-dependent manner. It is known that apoptosis is induced by the overexpression of Bak, which is recognized as a Bcl-2 antagonist. Moreover, P6 dampened the protein expression of the anti-apoptotic protein Bcl-2 in a concentration-dependent manner in HT-29 and DLD-1 cells (Figure 5A,B). Increased intracellular Ca^2+^ concentration is one of the hallmark events of cell apoptosis. Fluo-4 AM staining results showed that P6 produced a concentration-dependent increase in the intracellular Ca^2+^ concentration (Figure 5D,E). Additionally, mitochondrial Ca^2+^ overload induces an increase in intracellular ROS levels. Our results showed that P6 markedly increased intracellular ROS in both HT-29 and DLD-1 cells (Figure 5F,G). Together, these results revealed that P6 induced CRC cell apoptosis through Ca^2+^-mediated mitochondrial pathways. 

### 2.5. The p38-MAPK Signaling Pathway Mediates the Effect of P6 on Apoptosis in CRC Cells

**Figure 6:**
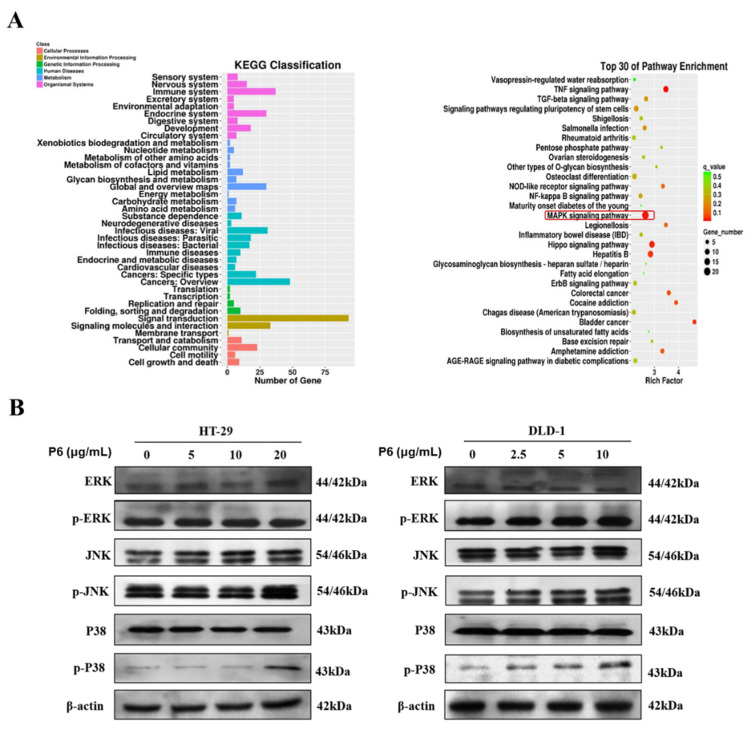
The p38-MAPK signaling pathway mediated the effect of P6 on apoptosis in CRC cells. (**A**) Total RNA was extracted from HT-29 cells treated with or without P6 and subjected to RNA sequencing. The histogram of differential genes in the KEGG classification and the scatter plot of differential genes from the KEGG enrichment analysis are shown, including the top 30 enriched KEGG pathways. (**B**) The effect of P6 on the MAPK pathway in HT-29 and DLD-1 cells with P6 treatment for 24 h.

To further explore the underlying mechanism by which P6 exerted pro-apoptotic effects, total RNA was extracted from the HT-29 cells treated with or without P6 for RNA-seq analysis. As illustrated in Figure 6A, the analysis of RNA-seq data revealed the KEGG classification and the top 30 pathways modulated by P6. Signal transduction, including the MAPK signaling pathway, TNF signaling pathway, and Hippo signaling pathway, is strongly influenced by P6 treatment in HT-29 cells. These signaling cascades have been identified to be involved in the cell apoptosis procedure [21,22,23]. Next, a Western blotting assay was performed to verify the effect of P6 on the MAPK signaling pathway in HT-29 and DLD-1 cells. The results demonstrated that P6 markedly augmented the phosphorylation of p38 in both CRC cell lines, indicating that P6 activated the MAPK signaling pathway (Figure 6B). However, P6 treatment had no obvious effect on the phosphorylation of ERK and JNK in both HT-29 and DLD-1 cells. Therefore, one might propose that the P6-induced mitochondrial apoptosis was partially via the activation of p38-mediated MAPK signaling pathways in CRC cells.

### 2.6. P6 Suppresses the Growth of CRC Tumor Xenografts In Vivo

**Figure 7:**
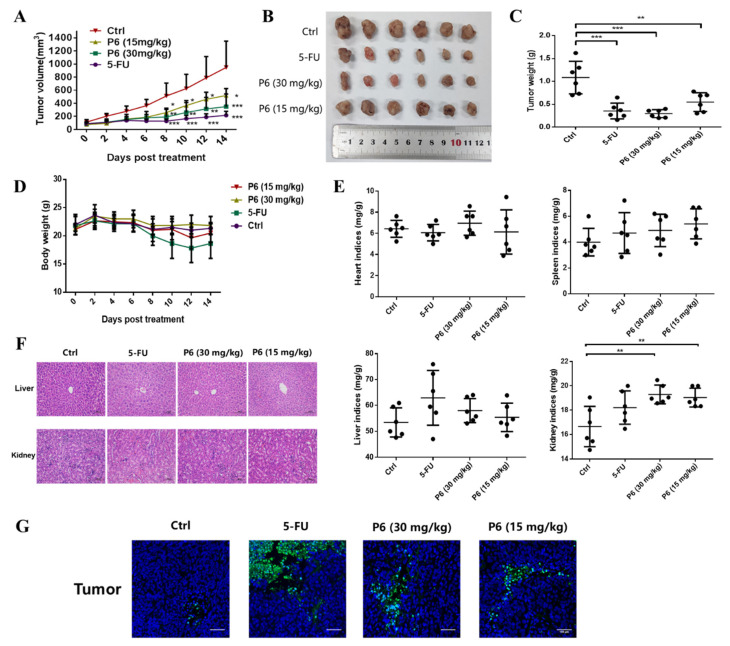
P6 suppressed the growth of HT-29 subcutaneous tumors in vivo. The antitumor activity of P6 was measured in xenograft nude mice. (**A**) Tumor volume curve. Results are expressed as mean ± SD; * *p* <0.05, ** *p* < 0.01 and *** *p* < 0.001 compared to the control group; *n* = 6 per group. (**B**) Photo collection of xenograft subcutaneous tumors in nude mice. (**C**) Tumor weight after 14-day administration of P6. Results are expressed as mean ± SD; ** *p* < 0.01 and *** *p* < 0.001, compared to the control group by one-way ANOVA and post hoc tests; *n* = 6 per group. (**D**) Body weight of the nude mice during drug treatment. (**E**) Organ index of nude mice. ** *p* < 0.05, compared with the control group. (**F**) The liver and kidneys of xenograft mice were histologically analyzed by H&E staining (scale bar = 50 μm). (**G**) TUNEL staining of tumor tissue (scale bar = 100 μm).

We further constructed a CRC HT-29 cell subcutaneous xenograft tumor model in BALB/c-nude mice to investigate the antitumor effect of P6 in vivo. The tumor volume (Figure 7A,B) and tumor weight (Figure 7C) of the P6-treated groups were significantly decreased compared to the model group after 14-day administration. According to record of tumor weight or tumor volume, P6 markedly inhibited the growth of subcutaneous tumors of HT-29 cells. As shown in Table 2, the tumor inhibition rates of the P6-treated groups (15 mg/kg and 30 mg/kg) reached 49.46% and 72.66%, respectively. Notably, the high dose P6-treated groups (30 mg/kg) showed an improved tumor inhibition rate compared to that of the 5-fluorouracil (5-FU)-treated group (67.28%). Moreover, the tumor growth curve showed that the tumor weight gradually decreased when the dose of P6 increased (Figure 7C). Meanwhile, body weight in each P6 treatment group showed a similar trend compared with the model group (Figure 7D). In addition, the organ indexes of the heart, spleen, liver, and kidney in the P6 treatment group showed no obvious difference when compared with the control group (Figure 7E). Hematoxylin and eosin (H&E) staining showed that, compared with the control group, there was no significant histological changes in the liver and kidneys in the P6 treatment group (15 and 30 mg/Kg). These results verified that P6 showed no obvious toxic side effects against mouse metabolic organs (Figure 7F). Furthermore, TdT-mediated dUTP nick-end labeling (TUNEL) staining demonstrated that the P6-treated groups could induce cell apoptosis in tumor tissues (Figure 7G).

## 3. Discussion

Colorectal cancer (CRC) kills nearly one million people each year and is becoming the world’s second most deadly cancer. As a consequence, CRC has produced a considerable disease burden and has become a major global public health problem [24]. At the present, 5-fluorouracil and oxaliplatin are still the first choice in the clinical chemotherapy treatment of CRC, including in the National Comprehensive Cancer Network (NCCN) Guidelines of CRC (version 2020.V1) [25]. However, the most serious treatment obstacle is the toxic side effects of 5-fluorouracil and oxaliplatin. For advanced CRC, there are only VEGFR receptor-targeted drugs (such as bevacizumab) and EGFR receptor-targeted drugs (such as cetuximab) on the market to date, which only have the effect of prolonging the approximately 3–5-months of survival for patients [26,27,28]. Additionally, due to their high treatment cost and significant side effects, these targeted drugs have greatly limited clinical applications [26,27,28]. Therefore, it is urgent to develop effective anti-CRC agents with low toxicity to effectively treat and prevent the recurrence and metastasis of CRC. Here, we report that P6, a novel peptide identified from the marine mollusk *Arca inflata*, was effective at suppressing CRC proliferation by inducing mitochondrial apoptosis (Figure 5). Moreover, P6 exhibited a prominent antitumor effect in the tumor xenograft model (Figure 7). Our study provides preliminary proof that P6 could be developed as a drug candidate for treating CRC.

Apoptosis, also known as programmed cell death, is the main mechanism for tumor cell death under the action of chemotherapeutic drugs [29]. An abnormal apoptosis signaling pathway, the loss of apoptosis signals, or the enhancement of anti-apoptosis signals can trigger a variety of pathological changes, leading to cancer or chemotherapy failure [30]. Under normal circumstances, apoptosis cannot trigger inflammation and the immune response; thus, promoting apoptosis has become an important strategy for tumor treatment [31]. There are two main apoptotic pathways in mammals, the death receptor pathway [32,33,34,35] and the mitochondrial pathway [36,37]. There is a cross between the two pathways, and certain signaling molecules can participate in different apoptotic pathways. In recent years, nearly a hundred peptides with antitumor activity have been discovered in marine organisms, of which more than 90% trigger apoptosis through targeted apoptotic mechanisms involving the mitochondria and death receptor pathways. Both apoptotic pathways require the mitochondrial-mediated activation of caspases. The mitochondrial pathway is the main mechanism of apoptosis induced by many antitumor drugs. The activation of mitochondrial apoptosis pathways is primarily due to several factors, including ROS production and Ca^2+^ overload in the cells [38]. When the Ca^2+^ homeostasis in tumor cells is disrupted, high levels of reactive oxygen species (ROS) are produced, resulting in the activation of cell apoptosis [38,39]. Interestingly, P6 showed a profound antitumor effect and induced tumor cell apoptosis by promoting ROS production and intracellular Ca^2+^ overload in CRC cells (Figure 5). It has been reported that Ca^2+^ overload could lead to mitochondrial dysfunction and mitochondrial ROS generation [39]. The underlying mode of action may include Ca^2+^-stimulated nitric oxide production, Ca^2+^ -stimulated increase in metabolic rate, Ca^2+^-induced cytochrome C (Cyt C) dissociation, Ca^2+^-induced opening of the permeability transition pore with the subsequent release of Cyt C, Ca^2+^-induced cardiolipin peroxidation, and Ca^2+^/calmodulin dependent protein kinase activation. Several marine peptides can change the Bcl-2/Bax ratio, activate caspases, and release Cyt C to induce cell apoptosis by stimulating Ca^2+^ overload and ROS production in tumor cells [40]. In this study, we found that P6 suppressed CRC cell proliferation (Figure 3) and induced cell apoptosis (Figure 4) by producing a concentration-dependent increase in intracellular Ca^2+^ concentration (Figure 5D,E). Additionally, mitochondrial Ca^2+^ overload induces an increase in intracellular ROS levels. Our results showed that P6 markedly reduced the mitochondrial membrane potential and increased intracellular ROS levels in both HT-29 and DLD-1 cells (Figure 5B,F,G). The decrease in mitochondrial membrane potential indicated that the permeability of the mitochondrial membrane was increased and the internal cytochrome C was released at the same time, which induced apoptosis. Furthermore, caspases are closely related to cell apoptosis. Many marine peptides induce tumor cell apoptosis by activating caspases. Pardaxin, a peptide consisting of 33 amino acid residues, is isolated from the secretions of a fish (*Pardachirus marmoratus*) found at the bottom of the Red Sea. Studies have shown that pardaxin can induce apoptosis in human fibrosarcoma HT-1080 cells, which is manifested by the increased activity of caspase-3, mitochondrial membrane potential changes, and the accumulation of reactive oxygen species (ROS) products [40]. P6 showed a similar effect, producing an increased activity of caspase-3 and mitochondrial membrane potential changes in CRC cells (Figure 5B,C). The subsequent Western blot results showed that P6 upregulated the expression of cytochrome C and the pro-apoptotic protein Bak (Figure 5C). It was further confirmed that P6 exerted an apoptosis-inducing effect through the mitochondrial pathway at the molecular level. 

Mitogen-activated protein kinases (MAPK) form a crucial signaling pathway that regulates cell fate decisions in response to external stimuli. Among them, p38 MAPK family members play an important role in regulating cell proliferation, senescence, and tumorigenesis [41]. Previous studies have shown that the altered expression of p38 proteins is often observed in CRC; thus, the drug-induced activation of the p38 MAPK cascade could be a scenario worth exploring for sensitizing CRC cells to apoptotic death [41]. Herein, we hypothesized that P6 might be a promising antitumor molecule via the activation of the p38 pathway. Considering the cell proliferation-suppressing and apoptotic effects of P6 (Figure 6A), we further investigated whether P6 influenced p38 MAPK pathways. As expected, P6 markedly increased the protein levels of the p38 proteins but showed no influence on the ERK and JNK pathways (Figure 6B), indicating the activation of the p38 MAPK cascades. Additionally, p38 activation-mediated apoptosis is sometimes induced by secondary routes, such as through the production of ROS [42]. In the present study, P6 suppressed proliferation and induced apoptosis in both HT-29 and DLD-1 cells. Hence, the effects of P6 on inducing mitochondrial apoptosis were partially dependent on ROS production.

## 4. Materials and Methods

### 4.1. Isolation and Identification of P6

*Arca inflata* Reeve materials were purchased from Qingdao of Shandong province, China; the production lot number was 20190410. The collected hemolymph of *A. inflata* was mixed with PBS in a ratio of 1:3 (*w*/*v*) and underwent ultrasound for 40 min at 4 °C. The supernatant was collected by 10,000 rpm centrifugation for 30 min at 4 °C. The supernatant was salted out with 100% ammonium sulfate, and the precipitate was collected by centrifugation (10,000 rpm for 30 min at 4 °C). The precipitate was dissolved in 20 mM Tris-HCl (pH 8.0) and dialyzed (molecular weight cut-off: 1 kDa) for 48 h. The samples were further separated by HPLC (Welch LP-C8, 5 μm, 4.6 × 250 mm). The sixth peak containing peptides was highlighted in red. Due to their similar hydrophobicity, the peptides in this marked peak were difficult to separate using a conventional approach. Hence, LC-MS/MS was applied to identify the peptides. The most abundant peptide fragment was named P6. According to the identified peptide sequences, the top 5 most abundant peptide fragments in this peak were synthesized by GenScript Co. (Shanghai, China) using a solid-phase peptide synthesis procedure. Their antitumor activities were tested using an MTT assay; only P6 possessed antitumor activity.

### 4.2. Purity of P6

High-performance liquid chromatography (HPLC) was applied to detect the purity of P6 using an Agilent series 1100 HPLC system connected to a ZORBAX®300SB-C8 column (4.6 × 150 mm, 5 µm; Agilent, Foster City, CA, USA). Water-trifluoroacetic acid (solvent A; 100:0.1, *v*/*v*) and acetonitrile-trifluoroacetic acid (solvent B; 100:0.1, *v*/*v*) were used as elution solvents. The elution procedure was set as follows: 55% solvent A and 45% solvent B with the flow rate at 1 mL/min. The wavelength of the UV detector was at 280 nm, and column temperature was 30 °C.

### 4.3. Molecular Weight Determination of P6

The precise molecular weight of P6 was determined using an ESI-MS spectrometer. The P6 sample was dissolved in distilled water and subsequently loaded into an API type 4000 QTRAP mass spectrometer (Applied Biosystems, Foster City, CA, USA). The mass spectrometer was used in the positive electrospray ionization (ESI + ve) mode. High-purity nitrogen gas was used for both drying (35 psi) and ESI nebulization (45 psi). The spectra were recorded over the mass/charge (*m*/*z*) range of 500–3000. 

### 4.4. De Novo Sequencing of P6 by Tandem Mass Spectrometry (MS)

LC-MS/MS was used to determine the amino acid sequence of P6. After P6 was separated by HPLC, it was analyzed by a TripleTOF 5600 LCMS (AB SCIEX, Concord, ON, USA) connected to a nanoscale liquid-chromatography (LC) system (NanoLC-Ultra 2D; Eksigent, Dublin, CA, USA). Briefly, a reversed-phase C18 column (75 μm × 15 cm, 3 µm, 120 Å; ChromXP, Eksigent, USA) was applied for the separation of the desalted hydrolyzed peptides. Mixtures of 0.1% (*v*/*v*) formic acid in 5% acetonitrile and 0.1% (*v*/*v*) formic acid in 95% acetonitrile were used as the mobile phases A and B, respectively. The elution procedure was as follows: 5–40% B for 65 min, 40–100% B for 10 min, and maintained for 5 min. The ionization voltage and capillary temperature were set as 2.3 kV and 150 °C, respectively. The molecular masses of the purified peptides were detected in MS/MS mode. The acquired data were further analyzed by PEAKS software version 8.5 (Waterloo, ON, Canada). The peptides with de novo scores greater than 85% were selected for use in the study.

### 4.5. Physicochemical Characterization of P6

Ultraviolet (UV) spectroscopy was used to detect whether P6 contained aromatic amino acids. UV-vis absorption spectroscopy was conducted with a UV-2450 UV-vis absorption spectrophotometer (Shimadzu, Osaka, Japan) equipped with a 1.0 cm quartz cell. P6 was dissolved in distilled water to prepare a 0.05 mg/mL solution, which was scanned in the wavelength range of 190–400 nm.

Infrared spectroscopy was used to analyze the functional groups of P6. P6 and potassium bromide were fully grinded and mixed, and the mixture was put into a mold for scanning by an EQUINOX55 FT-IR spectrometer (Bruker, Bremen, Germany). The scanning range was 400–4000 cm^−1^.

Circular dichroism was used to analyze the secondary structure conformation of P6. P6 was dissolved in distilled water to prepare a 0.05 mg/mL solution, and CD measurements were obtained using a Jasco J-810 spectropolarimeter (Japan Spectroscopic Co., Ltd., Hachioji, Tokyo, Japan) equipped with a 0.1 cm quartz cell. The average of eight scans was used to produce the final spectrum. The spectra were all corrected for solvent contributions. The scan speed was 50 nm/min, and the scan range was 260–190 nm. The other parameters were set as follows: response time of 0.5 s, bandwidth of 2 nm, data interval of 0.2 nm, and sensitivity of 20 mdeg. 

The three-dimensional structure of P6 was predicted using the PEP-FOLD3 server, https://bioserv.rpbs.univ-paris-diderot.fr/services/PEP-FOLD3/ (accessed on 24 December 2021). 

### 4.6. Reagents and Antibodies

N-acetyl-L-cysteine (A105420) was purchased from Aladdin (Shanghai, China). β-actin (1:1000, 3700), caspase-3 (1:1000, 9662), cleaved caspase-3 (1:1000, 9664), cleaved PARP (1:1000, 5625), Bcl-2 (1:1000, 5625), SAPK/JNK (1:1000, 9252), phospho-SAPK/JNK (1:1000, 4668), anti-rabbit IgG (1:4000, 7074S), anti-mouse IgG (1:4000, 7076S), Bak (1:1000, 12105), and cytochrome C (1:1000, 4280) were obtained from Cell Signaling Technology (Boston, Massachusetts, USA). The p38 MAPK (1:1000, AF6456), phospho-p38 MAPK (1:1000, AF3455), ERK1/2 (1:1000, AF0155), and phospho-ERK1/2 (1:1000, AF1014) were obtained from Affinity Biosciences (Pottstown, PA, USA). 

### 4.7. Cell Culture 

The human colorectal cancer cell lines (HT-29, DLD-1, HCT116, and SW620) and human normal liver cells (L02) were obtained from the Chinese Academy of Sciences Cell Bank of Type Culture Collection (Shanghai, China). The HT-29 and HCT116 cells were cultured in DMEM medium (Gibco, Gaithersburg, MD, USA) supplemented with 10% fetal bovine serum (FBS; Biological Industries, Beit Haemek, Israel), 100 units/mL penicillin, and 100 μg/mL streptomycin. The DLD-1 cells and SW620 cells were cultured in RPMI 1640 medium (Gibco, Gaithersburg, MD, USA) supplemented with 10% fetal bovine serum (FBS; Biological Industries, Beit Haemek, Israel), 100 units/mL penicillin, and 100 units/mL streptomycin. The cells were maintained in a humidified incubator with 5% CO_2_ at 37 °C.

### 4.8. Cell Viability Assay

The cytotoxicity of P6 was investigated using the 3-(4,5-dimethylthiazol-2-yl)-2,5-diphenyltetrazolium bromide (MTT) assay. L02 cells (2.5 × 10^3^ per well/100 μL), HT-29 cells, HCT116 cells, DLD-1 cells, and SW620 cells (1.5 × 10^3^ per well/100 μL) were seeded into 96-well plates containing DMEM or RPIM 1640 medium, respectively. After 24 h incubation at 37 °C and 5% CO_2_, the medium was replaced with various concentrations (0–27 μg/mL) of P6 for 48 h. Finally, 10 μL MTT solution (5 mg/mL) was added to each well, and the cells were incubated for 4 h. The MTT formazan product was dissolved in 200 μL DMSO and shaken for 10 min on the micro-oscillator. The optical density (OD) at 570 nm was recorded using a Synergy HT microplate reader (BioTek, Winooski, VT, USA). Each concentration was repeated at least three times. The inhibition rate was calculated as:

(%) = [1 − (D − D0)/(D1 − D0)] × 100%.

where D was the OD value of the experimental group, D0 was the OD value of the parallel solvent control group, and D1 was the OD value of the blank control group.

### 4.9. Colony Formation Assay

For the colony formation assays in monolayer cultures, the cells (1 × 10^3^ per well) were incubated in 6-well plates for 24 h. Two milliliters of the prepared P6 solutions at different concentrations were added, and 2 mL of complete medium was added to the control group. After continuous treatment for 14 days, the medium was discarded and the cells were gently rinsed 2–3 times with PBS buffer (1 mL/well). Then, 300 μL of 4% paraformaldehyde was added to each well, incubated for 25 min, and the 4% paraformaldehyde was aspirated. Five hundred microliters of 0.1% crystal violet solution were added to each well, incubated for 25 min, and then gently rinsed with PBS buffer 2–3 times. After fixation and staining, the colonies were imaged.

### 4.10. Cell Morphology Analysis

Hoechst 33342 staining measurements were performed with a Hoechst 33342 staining kit (C1022, Beyotime, Shanghai, China). The cells were seeded in confocal dishes at a cell density of 3 × 10^4^ cells/mL (200 μL/dish) and incubated at 37 °C and 5% CO_2_. After 24 h, the previous medium was discarded, and 200 μL of the prepared P6 solutions at different concentrations were added to the treatment group; 200 μL of complete medium was added to the control group. After 24 h of P6 treatment, the previous medium was aspirated and the cells were carefully rinsed 2–3 times. Two hundred microliters of Hoechst 33342 dye was added to each well, and after incubation in the dark for 15 min, the dye was discarded and the cells were carefully rinsed with PBS buffer 2–3 times. The photographs of the samples were recorded using a fluorescence microscope (Olympus, Tokyo, Japan).

### 4.11. Cell Apoptosis Assay

Cell apoptosis was detected using an Annexin V-FITC/PI staining kit (FXP018-100, 4A Biotech, Beijing, China). Cells (1 × 10^5^ per well) were seeded into 6-well plates and incubated for 24 h with various concentrations of P6 (0, 5, 10, and 20 μg/mL for HT-29 cells or 0, 2.5, 5, and 10 μg/mL for DLD-1 cells) for 24 h. Then, the cells were washed, trypsinized, harvested, suspended in binding buffer, and stained with Annexin V-FITC and PI following the manufacturer’s instructions. The suspended cells were incubated with FITC and PI in the dark at room temperature for 15 minutes. The samples were immediately analyzed by flow cytometry (BD FACSCanto, Canto, NJ, USA).

### 4.12. Cell Cycle Assay

Cells (1 × 10^5^ per well) were seeded into 6-well plates and incubated for 24 h with various concentrations of P6 (0, 5, 10, and 20 μg/mL for HT-29 cells or 0, 2.5, 5, and 10 μg/mL for DLD-1 cells) for 24 h. The cells were harvested and suspended in 70% ethanol. Then, the samples were stored at −20 °C for 2 h and stained with PI for 15 min in the dark at room temperature. The samples were immediately analyzed by flow cytometry (BD FACSCanto, Canto, NJ, USA).

### 4.13. Mitochondrial Membrane Potential (MMP) Assay

The mitochondrial membrane potential of colorectal cancer cells was determined using a JC-1 staining kit (C2006, Beyotime, Shanghai, China) with flow cytometry. Colorectal cancer cells were seeded into a 6-well plate at a density of 1 × 10^5^ cells per well. After incubation overnight, the cells were treated with different concentrations of P6 (0, 5, 10, and 20 μg/mL for HT-29 or 0, 2.5, 5, and 10 μg/mL for DLD-1) for 24 h. Next, the cells were washed, trypsinized, harvested, and stained with JC-1 working buffer at 37 °C for 20 min in the dark. The samples were washed three times with PBS and immediately analyzed by flow cytometry (BD FACSCanto, Canto, NJ, USA).

### 4.14. Intracellular ROS Detection

A DCFH-DA staining kit (S0033, Beyotime, Shanghai, China) was used to detect intracellular ROS in CRC cells. Briefly, cells (1.5 × 10^5^ per well) were seeded into a 6-well plate and incubated at 37 °C and 5% CO_2_ for 24 h. Then, each well was covered with various concentrations of P6 (0, 5, 10, and 20 μg/mL for HT-29 or 0, 2.5, 5, and 10 μg/mL for DLD-1) for 24 h. Next, the cells were washed with pre-cooled PBS, trypsinized, and harvested. Finally, the cells were stained with DCFH-DA at 37 °C for 30 min. The samples were washed three times with medium without serum and immediately analyzed by flow cytometry (BD FACSCanto, Canto, NJ, USA).

### 4.15. Intracellular Ca^2+^ Detection 

Intracellular Ca^2+^ was detected using a Fluo-4 AM staining kit (S1060, Beyotime, Shanghai, China) and analyzed by flow cytometry. Colorectal cancer cells were seeded into a 6-well plate at a density of 1 × 10^5^ cells per well. After incubation overnight, the cells were treated with different concentrations of P6 (0, 5, 10, and 20 μg/mL for HT-29 cells or 0, 2.5, 5, and 10 μg/mL for DLD-1 cells) for 24 h. Next, the cells were washed, trypsinized, harvested, and stained with Fluo-4 AM at 37 °C for 30 min in the dark. The samples were washed three times with PBS and immediately analyzed by flow cytometry (BD FACSCanto, Canto, NJ, USA).

### 4.16. Western Blotting Analysis

HT-29 and DLD-1 cells were seeded into 6-well plates (5 × 10^5^ per well) and incubated in medium for 24 h, after which the medium was replaced with various concentrations of P6 (0, 5, 10, and 20 μg/mL for HT-29 cells or 0, 2.5, 5, and 10 μg/mL for DLD-1 cells) for 24 h. Then, the cells were washed three times with pre-cooled PBS and lysed with RIPA buffer (P0013K, Beyotime, Shanghai, China) on ice. Protein samples were collected and boiled for 15 min. The concentrations of the protein samples were quantified using a BCA protein assay kit (P0012S, Beyotime, Shanghai, China). Equal amounts of denatured protein samples were separated on 10% to 12% (*w*/*v*) sodium dodecyl sulfate (SDS)-polyacrylamide gels and transferred to polyvinylidene fluoride (PVDF) membranes (Bio-Rad, Hercules, CA, USA). The membranes were blocked in 5% BSA at room temperature for 2 h and incubated with primary antibodies overnight at 4 °C. Then, the membranes were incubated with horse radish peroxidase-linked secondary antibodies at room temperature for 2 h. After they were washed three times with TBST, the membranes were exposed to X-ray film to detect the expressions of the target proteins, which were enhanced using the ECL Kit (Tanon, Shanghai, China).

### 4.17. RNA-Seq Analysis

HT-29 cells in the logarithmic growth phase were collected and passaged in 100 mm Petri dishes. When the cell growth covered approximately 70% of the Petri dishes, the P6 solution or medium solution was added. After 24-hour incubation, the previous medium was discarded. The cells were carefully rinsed twice with pre-cooled PBS buffer and 3 mL of TRIZOL lysate was added to each group, lysed on ice for 5 min, and the cell lysates were collected. The purified total RNA from HT-29 cells treated with P6 or without P6 was extracted and sent to Shanghai Bohao Biotech. Co., Ltd. (SHBIO, Shanghai, China) for RNA-seq analysis using an Illumina HiSeq system after transcription. The quantity of gene expression was calculated by FPKM (fragments per kilobase of transcript per million fragments mapped). Genes with log2 (fold change) > 1 and Q < 0.001 were considered as differentially expressed genes (DEGs). Gene cluster analyses and enriched KEGG (Kyoto Encyclopedia of Genes and Genomes) pathway analyses were performed based on the DEGs. 

### 4.18. In Vivo Studies

All animal experiments were approved by the Medical Laboratory Animal Center of Jinan University and followed the ethical requirements for laboratory animals. Male BALB/c-nude mice were obtained from Beijing Huafukang Biotechnology Co. Ltd. These mice were fed breeding feed and water in an SPF laboratory at room temperature. HT-29 cells (1.5 × 10^6^ cells in 0.1 mL phosphate-buffered saline) were subcutaneously injected into the left armpit of 6-week-old male BALB/c nude mice. Tumor volume and body weight were measured every 2 days, and tumor volume was calculated using the following formula: (short diameter)^2^ × (long diameter)/2. When the tumor volume reached about 100 mm^3^, these mice were randomly divided into four groups (*n* = 6 in each group): the model group, 5-FU treatment group, P6 (30 mg/kg) treatment group, and P6 (15 mg/kg) treatment group. The reference drug (5-FU) and P6 were dissolved in normal saline and administered by subcutaneous injection at a volume of 0.1 mL/10g. After 14 days of administration, all mice were anesthetized by intraperitoneal injection of sodium pentobarbital (1 mg/20 g mouse) and euthanized. The organs were obtained for immunohistochemical experiments.

### 4.19. Histopathologic Examination and TUNEL Staining

The organs were fixed in 10% buffered paraformaldehyde solution and sectioned at 4 μm thicknesses. Hematoxylin and eosin (H&E) was used to stain the specimens. An optical microscope (Nikon, Tokyo, Japan) was used to observe the histopathological changes in the specimens.

A TUNEL assay was performed according to the manufacturer’s instructions using a one-step TUNEL apoptosis assay kit (C1086, Beyotime, Shanghai, China). TUNEL-positive tissues or cells were imaged under a fluorescence microscope (Olympus, Tokyo, Japan). 

### 4.20. Statistical Analysis 

The differences between two groups were analyzed by the two-tailed Student *t*-test, and comparisons of multiple groups were carried out using one-way analysis of variance (ANOVA). The data represent the mean ± standard deviation (SD) in all figures. *p* < 0.05 was chosen as the level of significant differences.

## 5. Conclusions

In summary, we identified a novel peptide termed P6 from the marine mollusc *Arca inflata* and demonstrated its anti-CRC effect in vivo and in vitro. Our results further indicated that P6 promoted Ca^2+^ influx, a reduction in mitochondrial membrane potential, and the generation of ROS, and thereby induced apoptosis in colon cancer cells. Additionally, the MAPK pathway may contribute to the induction of apoptosis. These findings suggest that P6, which functions as a positive regulator of apoptosis, may be a potential molecule for acquiring therapeutic effects in CRC cells, and warrants further study in the future.

## Figures and Tables

**Table 1:** The IC_50_ values of P6 against colorectal cancer and normal cell lines.

Cell Lines	IC_50_ (μg/mL)	
	P6	DDP
HT-29	4.43 ± 0.15	1.90 ± 0.61
DLD-1	2.14 ± 0.28	1.06 ± 0.41
HCT116	10.88 ± 0.72	1.38 ± 0.20
SW620	>27	1.08 ± 0.12
L02	>150	/

DDP—cis-diamminedichloro-platinum II.

**Table 2:** Effect of P6 on tumor inhibition rate (%) of HT-29 xenograft mice.

Group	Dose	Tumor Weight (g)	Tumor Inhibition Rate (%)
Saline (Ctrl)	0.1 mL/10 g	1.09 ± 0.36	/
5-FU	25 mg/kg	0.36 ± 0.17 ***	67.28
P6 (high dose)	30 mg/kg	0.30 ± 0.09 ***	72.66
P6 (low dose)	15 mg/kg	0.55 ± 0.20 **	49.46

Values represent the mean ±SD. ** *p* < 0.01 and *** *p* < 0.001, compared with the model group; *n* = 6.

## Data Availability

All data generated or analyzed during this study are included in this published article.
